# Enhancing the Nutritional Value and Sensory Quality of Traditional Saudi Kleija Through Partial Substitution of Wheat Flour with African Hulu-Mur Flakes Powder

**DOI:** 10.3390/foods15193535

**Published:** 2026-10-03

**Authors:** Rehab F. M. Ali, Ayman M. El-Anany

**Affiliations:** 1 Department of Food Science and Human Nutrition, College of Agriculture and Food, Qassim University, Buraydah 51452, Saudi Arabia; 2 Special Food and Nutrition Department, Food Technology Research Institute, Agricultural Research Center, Giza 12619, Egypt; aymanelanany82@gmail.com

**Keywords:** Saudi Kleija, Hulu-mur flakes powder, ethnic foods, diet supplementation, antioxidants, sensory evaluation

## Abstract

This study presents the first investigation into the potential of integrating Sudanese Hulu-mur flakes powder (HMFP), a fermented sorghum product rich in bioactive compounds, into Saudi Kleija, a traditional pastry. White wheat flour was partially substituted with 2, 4, 6, 8, 10, and 12% *w*/*w* of HMFP to produce the developed Kleija samples. Proximate composition, minerals, total phenolics, total flavonoids, total carotenoids, DPPH radical scavenging activity percentage, and sensory characteristics of the Kleija samples were examined. The inclusion of HMFP resulted in significant (*p* ≤ 0.05) improvements in the functional properties of the composite flours. Incorporation of HMFP significantly (*p* ≤ 0.05) enhanced the nutritional profile, particularly in protein, ash and fiber content. The highest ash content (3.29%) and fiber levels (7.69%) were recorded for samples with 12% HMFP, while energy content decreased from 463.60 to 459.14 kCal/100 g at 12% HMFP. Total phenolics, flavonoids, carotenoids, and DPPH radical scavenging activity were 2.42, 2.33, 1.50, and 1.54 times higher, respectively, in Kleija with 12% HMFP compared to the control, demonstrating dose-dependent enhancement of antioxidant capacity. Acceptability scores of 8.37, 8.45, and 8.49 at 8%, 10%, and 12% HMFP indicated strong consumer preference. This work demonstrates a successful model for cross-cultural food innovation that enhances nutritional quality while maintaining sensory acceptability, positioning HMFP–Kleija as a nutritionally enhanced traditional food product with significant commercial potential.

## 1. Introduction

Traditional ethnic foods are a vital repository of cultural heritage and a significant source of functional ingredients that enhance dietary health. In Arab countries, staples such as dates are rich in dietary fiber, polyphenols, and antioxidants, supporting gastrointestinal health and mitigating oxidative stress [1,2]. Fermented products like Laban and Kishk provide probiotics and bioactive peptides that bolster gut health and immune function [3,4]. Furthermore, the pervasive use of herbs and spices delivers notable anti-inflammatory and antimicrobial benefits [5,6], while whole grains like burghul and freekeh offer high levels of fiber and minerals linked to improved metabolic health [7]. This intersection of culture and nutrition makes traditional foods a compelling focus for dietary innovation.

The integration of traditional foods into functional food development represents a growing paradigm in food science, emphasizing the preservation of cultural heritage while addressing contemporary nutritional challenges [8,9]. This approach aligns with the concept of ‘food heritage innovation,’ which posits that traditional foods can serve as vehicles for dietary intervention when appropriately modified. The present study was designed with three main objectives: (1) to evaluate the effect of HMFP incorporation on the proximate composition, mineral content, and bioactive compound profile of Kleija, (2) to determine the techno-functional properties (water and oil absorption, emulsifying, and foaming capacities) of the composite flours, and (3) to determine the optimal substitution level that achieves the greatest nutritional improvement without negatively affecting consumer acceptability.

Among these traditional foods, Kleija stands out as a cherished pastry from the Qassim region of Saudi Arabia, reflecting the area’s rich culinary identity. It is characterized by a crisp exterior and a chewy, spiced filling, typically prepared from a dough of wheat flour, sugar, milk, and fat [10,11,12]. Recent innovations have focused on enhancing its nutritional profile, such as replacing refined sugar with date-based sweeteners, which successfully maintain sensory qualities while adding nutritional benefits [11]. Beyond its role as a snack, Kleija is a symbol of cultural expression, underscored by community celebrations dedicated to it [12,13].

**Figure 1:**
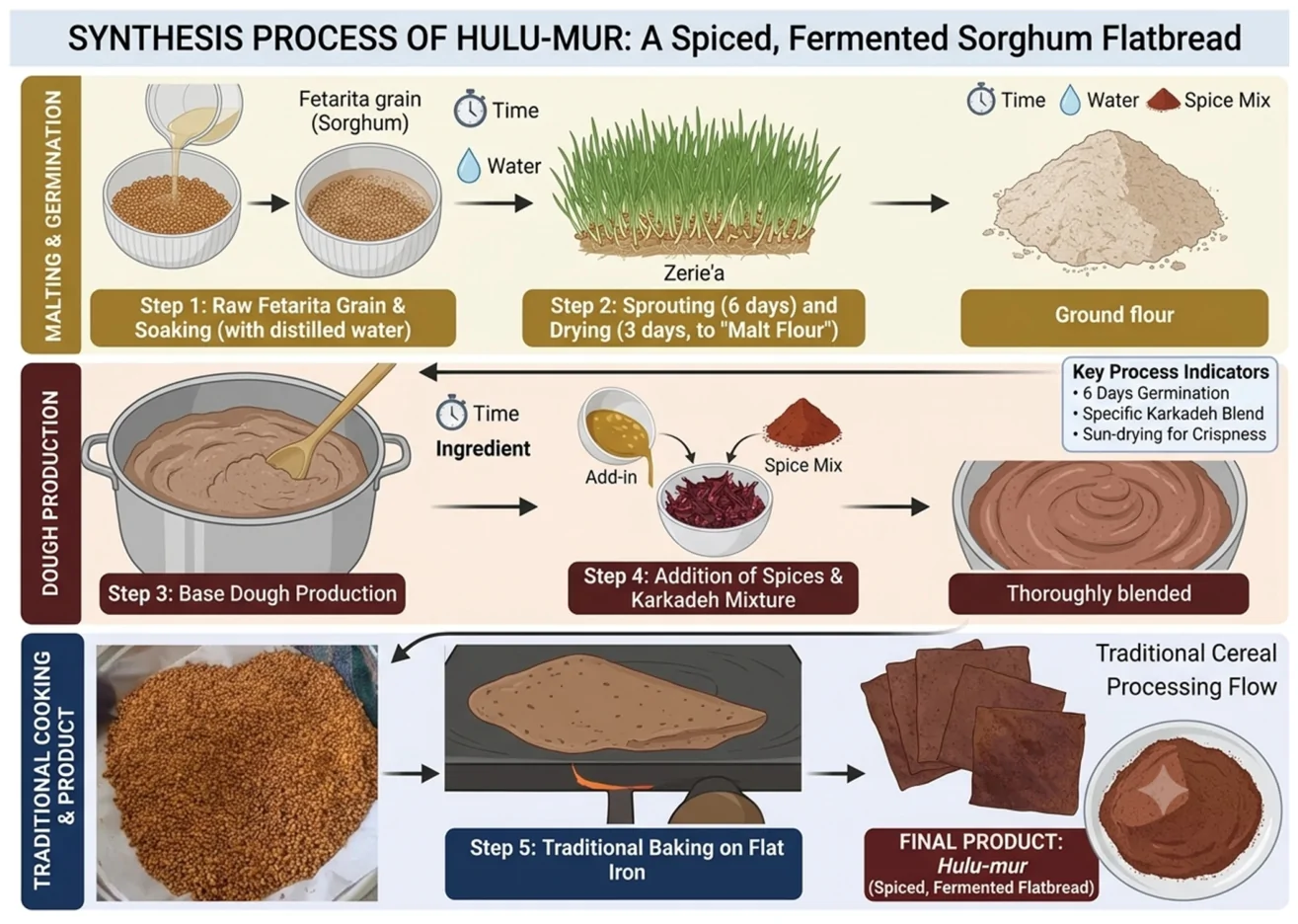
Traditional processing steps of Sudanese Hulu-mur flakes.

Simultaneously, in Sudan, Hulu-mur represents another pillar of traditional food culture, a non-alcoholic beverage made from fermented sorghum flour, particularly the fetarita variety. Its preparation is an intricate process involving malting, fermentation, and baking into thin sheets that are broken into flakes [14]; see Figure 1. This fermentation, driven by lactic acid bacteria and yeasts, is crucial as it not only develops the unique flavor but also significantly enhances the nutritional value by increasing the bioavailability of phenolic compounds and boosting antioxidant activity [15,16]. The inclusion of ingredients like tamarind, dates, and hibiscus further enriches its profile of antioxidants and micronutrients, solidifying Hulu-mur’s status as a functional food [14].

While both Kleija and Hulu-mur are culturally significant in their own right, their potential combination in a combined product remains completely unexplored. This represents a significant gap in the scientific literature, as no studies have investigated the techno-functional compatibility or nutritional enhancement potential of integrating a fermented sorghum product into a wheat-based pastry matrix. Therefore, this study aims to develop a novel, nutritionally enhanced food product by integrating Sudanese Hulu-mur flakes powder into Saudi Kleija and to evaluate its resulting nutritional, functional, and sensory properties.

## 2. Material and Methods

Hulu-mur flakes, a traditional fermented sorghum product, were purchased from a local market in Khartoum, Sudan. Whole wheat flour (100% extraction rate) and white wheat flour (70% extraction rate) were obtained from First Mills Co. (Buraydah, Qassim, Saudi Arabia). Both flours were derived from hard red winter wheat (Triticum aestivum), not durum wheat. The white wheat flour had an approximate protein content of 10.5–11.5% (14% moisture basis), and the whole wheat flour had an approximate protein content of 12.0–13.0% (14% moisture basis), as determined by the manufacturer using AACC Method 46–30. Both flours were from the 2024 harvest season and were stored at room temperature (25 ± 2 °C) in airtight containers until use. Cold-pressed extra virgin olive oil, fine sugar, full-cream milk powder, fresh egg, salt, citric acid, cinnamon powder, cardamom powder, black lemon powder, and Khalas date molasses were obtained from a local market in Buraydah, Qassim, Saudi Arabia. All of the chemicals used in the present study were of analytical grade. All experiments were performed in accordance with the relevant guidelines and regulations of Food Technology Research Institute, Agricultural Research Center, Egypt.

### 2.1. Preparation of Hulu-Mur Flakes Powder

Commercially available dried Hulu-mur flakes (moisture content 7.6%) were purchased. These flakes are a traditional fermented product made from sorghum (Sorghum bicolor, fetarita variety). The flakes were ground directly in an electric grinder (Panasonic, Mixer Grinder 1 L 1000 W MX-AC210 SWTZ, Panasonic Corporation, Kadoma, Osaka, Japan). To maintain a consistent particle size, the obtained powder was passed through a 0.5 mm sieve. The prepared Hulu-mur flakes powder (HMFP) was packed in clean, low-density polyethylene bags and stored in a refrigerator (4 ± 1 °C) for further use.

### 2.2. Techno-Functional Properties of White Wheat Flour and Hulu-Mur Flakes Powder (HMFP) and Composite Flours

The procedures that were advised by Sosulski [17] and Sosulski et al. [18] were implemented to determine the water and oil absorption capacities, respectively, of white wheat flour (WWF), HMFP, and the composite flours (WWF with 2–12% HMFP). The methods of Lawhon et al. [19] and Neto et al. [20] were used to estimate the foaming and emulsifying capabilities, respectively, of the same flour samples. Although these properties are not directly utilized in Kleija production (which does not involve whipping or emulsifying steps), they were measured to characterize the surface-active properties of HMFP, which may influence dough handling and final product quality.

### 2.3. Preparation of the Filling Flavored Mixture

To prepare 100 g of the flavored mixture, 24.5 g of ground black lemon, 24.5 g of ground cinnamon, 24.5 g of ginger, 24.5 g of ground cardamom, and 2 g of citric acid were combined. Fifty grams of the obtained spice mixture was then blended with 1 kg of fine sugar to obtain the final flavored sugar mixture. This flavored sugar mixture was mixed at a ratio of 1:1 (*w*/*w*) with Khalas date molasses.

### 2.4. Preparation of Kleija Samples

The control Kleija sample (0% HMFP) was prepared from a base formula comprising a blend of flours. Specifically, 5 kg of whole wheat flour (100% extraction rate) and 5 kg of white wheat flour (70% extraction rate) were mixed with 400 g of full-cream milk powder, 400 g of fine sugar, 30 g of salt, 90 g of cardamom powder, and 90 g of ginger powder. This dry mixture was combined with 2.5 L of cold-pressed extra virgin olive oil in a mixing bowl for 10 min.

**Figure 2:**
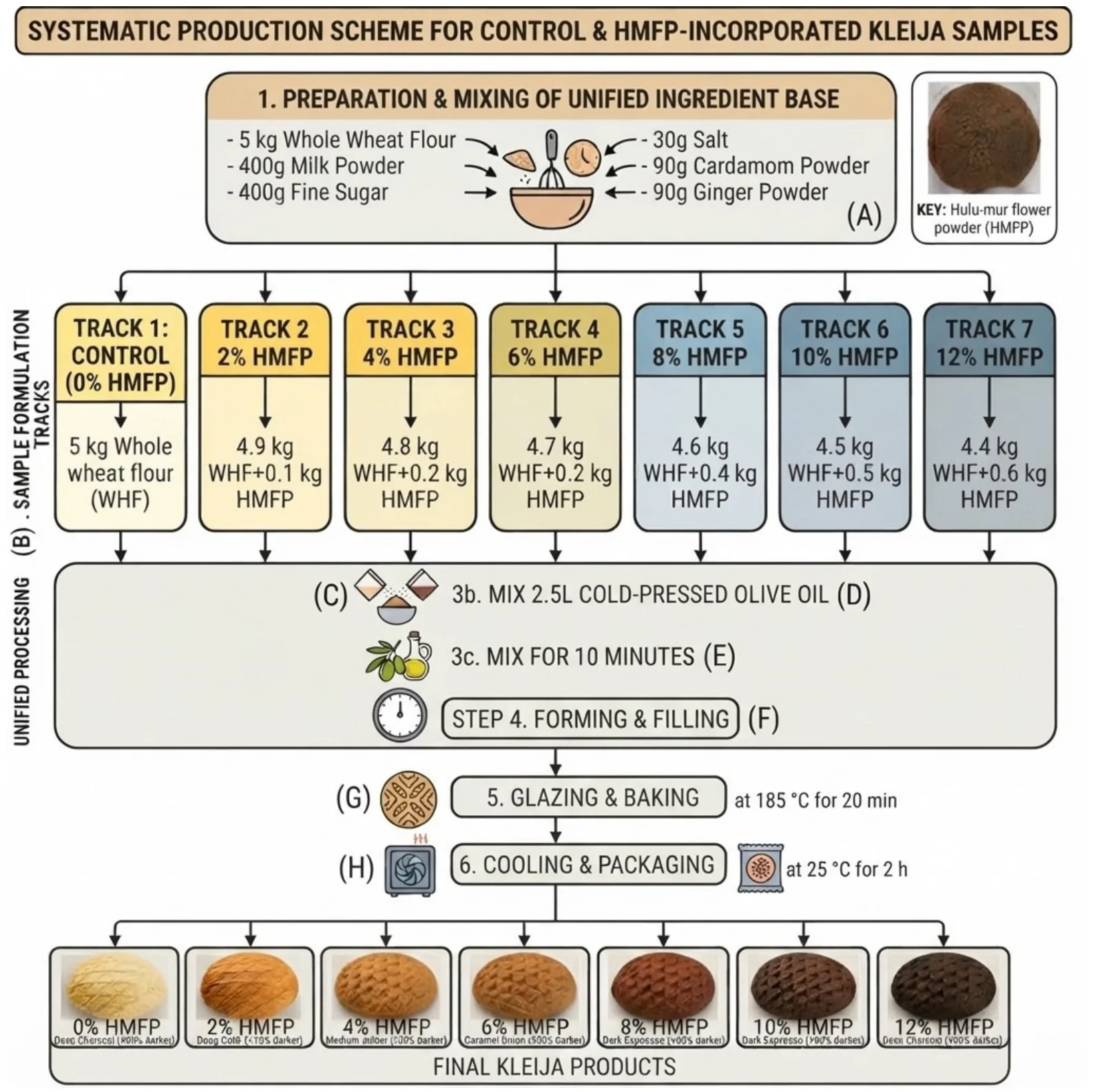
Systematic production scheme for the preparation of control and HMFP-incorporated Kleija samples. The flow diagram illustrates the sequential steps from ingredient preparation to final baked product, including: (**A**) preparation of dry ingredients (flour blend, sugar, milk powder, salt, spices), (**B**) mixing with oil and incremental water addition to form dough, (**C**) dough resting, (**D**) portioning (50 g) and filling with flavored sugar–molasses mixture, (**E**) shaping into traditional Kleija balls with characteristic pattern stamping, (**F**) egg glazing, (**G**) baking at 185 °C for 20 min, and (**H**) cooling at 25 °C for 2 h. The substitution levels of HMFP (2–12% *w*/*w*) were incorporated at the flour blending stage.

Thereafter, cold water (5 °C) was added incrementally to the dry mixture with continuous mixing at low speed (60 rpm) for 2 min, followed by medium speed (120 rpm) for 8 min, using a planetary mixer (Kenwood KM010, Kenwood Ltd., Havant, UK). Water addition was terminated once the dough reached the desired consistency, characterized by a smooth, non-sticky, and elastic texture. The total water added for the control sample was 5 L. For HMFP-fortified samples, due to the higher water absorption capacity of HMFP, the amount of cold water was adjusted accordingly to 5.31, 5.53, 5.81, 5.96, 6.11, and 6.14 L for the 2% to 12% HMFP samples, respectively. The water addition levels for each formulation were determined in preliminary trials based on maintaining constant dough consistency, measured using a dough rheometer (Brabender Farinograph, Duisburg, Germany), targeting a consistent farinograph consistency of 500 Brabender Units (BU) across all formulations. The specific water adjustments were determined empirically to achieve this target consistency. The mixed dough was rested at a controlled room temperature of 25 °C for 30 min. It was then divided into 50 g portions, each filled with 10 g of the flavored sugar–molasses mixture. The filled dough was shaped into traditional Kleija balls, stamped with a characteristic pattern, and the surface was glazed with 1 g of whipped egg. The prepared Kleija were left to stand for 10 min before baking. Baking was carried out in a deck oven (MIWE Condo, Arnstein, Germany) at 185 °C for 20 min. After baking, the samples were cooled in a cooling cabinet at 25 °C for 2 h, packed in polyethylene bags, and subjected to sensory evaluation after a total cooling time of 3 h. A systematic overview of the production process for both the control and experimental samples is provided in Figure 2.

For the experimental samples, the white wheat flour in the base formula was partially replaced with Hulu-mur flakes powder (HMFP) at levels of 2, 4, 6, 8, 10, and 12% (*w*/*w*). All other ingredients and procedures remained identical to the control. Due to the higher water absorption capacity of HMFP, the amount of cold water was adjusted accordingly to 5.31, 5.53, 5.81, 5.96, 6.11, and 6.14 L for the 2% to 12% HMFP samples, respectively. These specific substitution levels and water adjustments were determined based on preliminary tests.

### 2.5. Chemical Composition

Moisture content (AOAC 925.09 B), crude protein (AOAC 950.36), crude fat (AOAC 960.39), ash (AOAC 930.22), and total dietary fiber (TDF) were determined using AOAC method 991.43 following the AOAC methods [21] for the baked Kleija samples. The carbohydrate content was calculated by difference, subtracting the percentages of moisture, crude protein, ash, ether extract, and total dietary fiber from 100%. Results were expressed as grams per 100 g of dry matter, as the average of five replicates. The energy value (EV), expressed in kcal/100 g, was calculated as follows: EV (kcal/100 g) = % proteins × 4.0 + % available carbohydrates × 4.0 + % lipids × 9.0 [22].

### 2.6. Mineral Content

A total of 0.5 g of the sample (baked Kleija samples), ground to pass through a 0.5 mm sieve using an electric grinder (Panasonic, Mixer Grinder 1 L 1000 W MX-AC210 SWTZ) was wet-digested for 3 h with a 2:1 ratio of HNO_3_ and HClO_4_ on a heated mantle [23]. Digested samples were filtered through a 0.45 μm Millipore filter and diluted with 100 mL of distilled water. Ca, Mg, Fe, Cu, Mn, and Zn concentrations were determined using an Atomic Absorption Spectrophotometer (Hitachi Zeeman Japan Z-8000, Hitachi High-Technologies Corporation, Tokyo, Japan) equipped with standard cathode lamps and air acetylene flames. Na and K concentrations were determined using a Jenway PFP7 Flame Photometer (Cole-Parmer Ltd., Staffordshire, UK). Phosphorus content was assessed using the colorimetric technique provided by Winiarska-Mieczan and Kwiecien [24].

### 2.7. Phenolic Compounds Extraction

The extraction process involved shaking 0.6 g of the material (baked Kleija samples, previously freeze-dried using a Christ Alpha 1–4 LDplus freeze dryer and ground to pass through a 0.5 mm sieve) in 30 mL of ethanol 80% for 120 min using an electric shaker (Thermoline, Wetherill Park, NSW, Australia). The extracts were centrifuged for 15 min at 4500 rpm and 1050× *g* (Thermo Fisher Scientific, Dreieich, Germany). The supernatant was decanted and kept at −20 °C for future analysis.

### 2.8. Determination of Total Phenolic Content (TPC)

The Folin-C Assay was used to determine total phenolic content (TPC), following the techniques established by Kupina et al. [25]. TPC was calculated as milligrams of gallic acid equivalent per gram dry weight.

### 2.9. Determination of the Total Flavonoid Content (TFC)

TFC was determined using the aluminum chloride calorimetric method described by Alsayari et al. [26]. TFC is measured in mg of catechin equivalents (CE) per gram of dry weight (DW).

### 2.10. Determination of Total Carotenoids

Total carotenoids (mg/g) were measured using the methods described by Abdel-Moatamed et al. [27].

Total carotenoids (mg/g) = 1000 × A470 − [2.860 × Ca − 129 × Cb]/245. Ca denotes chlorophyll a concentration (mg/g) at 666 nm, while Cb represents chlorophyll b concentration (mg/g) at 653 nm.

### 2.11. 2,2-Diphenyl-1-picrylhydrazyl Radical Scavenging Activity Assay

2,2-diphenyl-1-picrylhydrazyl (DPPH) radical scavenging activity of ethanolic extracts was assessed using the procedures described by Mohammed et al. [28].

A total of 5 mL of a 6 × 10^−3^ M methanolic solution of the DPPH radical was combined with an aliquot (100 mL) of ethanolic extract. After an adequate shake, the mixture was allowed to stand at room temperature for 30 min in the dark. With a UNICO UV/VIS-2100 A spectrophotometer (UNICO UV/VIS-2100A, UNICO, Dayton, NJ, USA), the absorbance at 517 nm was measured in comparison to a blank. Using the following formula, the percentage of inhibition was determined:

(%) = [(Abs control − Abs sample)/Abs control] × 100

where Abs control is the absorbance of DPPH radical + methanol; Abs sample is the absorbance of DPPH radical + sample extract/standard.

### 2.12. Sensory Evaluation

Sensory evaluation was conducted using a randomized complete block design with panelists as the blocking factor. Each panelist evaluated all seven samples (control and six HMFP-substituted formulations) in a single session. To minimize expectation bias, samples were presented monadically in a randomized order using a Latin square design, ensuring that each sample appeared in each position approximately equal numbers of times. Panelists were blinded to sample identity; samples were coded with random three-digit numbers and served in identical white plates. No information about the formulations was provided to panelists prior to or during evaluation. To minimize position bias and carryover effects, a 2 min inter-stimulus interval was enforced between samples, during which panelists cleansed their palates with distilled water and unsalted crackers. Panelists were also instructed to take a 5 min rest break after evaluating the fourth sample to reduce sensory fatigue. The total number of samples per session was limited to seven, consistent with standard sensory evaluation guidelines recommending no more than seven samples per session to avoid fatigue effects. The sensory evaluation was conducted in a temperature-controlled (25 °C) and humidity-controlled (50–55% RH) sensory evaluation room under daylight-mimicking fluorescent lighting. Individual booths were used to prevent panelist interaction and social influence, and panelists were instructed not to discuss their evaluations with other panelists. A 9-point hedonic scale (1 = ‘dislike extremely’; 9 = ‘like extremely’) was used to rate appearance, exterior color, interior color, texture, taste, odor, and overall acceptability [11]. Attributes were evaluated in a fixed order, and panelists were provided with water to cleanse their palates between samples. The evaluation protocol was reviewed and approved by the Health and Bioethics Research Ethics Committee, Qassim University (Approval No. 26-15-6), and all participants provided written informed consent prior to participation.

### 2.13. Statistical Analysis

SPSS (version 20) was used to analyze the data. All analyses were performed on samples obtained from five independent production batches for each Kleija formulation, with each batch analyzed in duplicate. The data were statistically analyzed with five independent replicates (n = 5), representing separate production batches, except for sensory evaluation results (n = 50). Data were analyzed for variance and means were separated using Duncan’s Multiple Range Test with 95% confidence.

### 2.14. Figure Generation

All figures were generated using Microsoft Excel 2019 (Microsoft Corporation, Redmond, WA, USA) and GraphPad Prism version 9.0 (GraphPad Software, San Diego, CA, USA).

## 3. Results and Discussion

### 3.1. Techno-Functional Properties of White Wheat Flour, Hulu-Mur Flakes Powder (HMFP) and Composite Flours

Table 1 shows the water absorption, oil absorption, emulsifying and foaming capacities of white wheat flour, Hulu-mur flakes powder (HMFP) and the composite flours. The water absorption capacity (WAC) of flour is a critical parameter influencing its functional properties, particularly in composite flours [20]. WAC values ranged from 1.40 to 2.33 g water/g sample (Table 1). HMFP exhibited the highest WAC at 2.33 g water/g sample, which is consistent with the high fiber content of sorghum-based products that enhances water retention through hydrogen bonding and capillary action within the fiber matrix, as previously reported for fiber-rich ingredients [29,30]. The incorporation of HMFP into white wheat flour resulted in a progressive increase in WAC with increasing substitution levels. The WAC values for white wheat flour enriched with HMFP at 2%, 4%, 6%, 8%, 10%, and 12% were found to be 1.42, 1.44, 1.46, 1.49, 1.51, and 1.53 g H_2_O/100 g, respectively, representing increases of 1.01- to 1.09-fold compared to the control sample (Table 1). This observed increase suggests that the composite flours’ capacity to absorb water improved with higher HMFP levels (*p* ≤ 0.05), which is important for dough formation and product quality [31]. The observed increase in WAC with HMFP substitution has practical implications for dough processing and product quality. Higher WAC values indicate that HMFP-supplemented flours require more water to achieve optimal dough consistency, which was reflected in our experimental formulation adjustments (from 5.0 L for the control to 6.14 L for 12% HMFP). This increased water absorption can lead to improved dough yield, enhanced moisture retention during baking, and potentially softer texture in the final product. However, excessive water absorption may also affect dough handling properties and require adjustments in mixing time or dough resting time. The WAC values observed in this study (1.40–2.33 g water/g sample) are consistent with those reported for other fiber-rich composite flours [32,33] and suggest that HMFP can be effectively incorporated into Kleija formulations with appropriate water adjustments. Similar studies have shown that composite flours, such as those incorporating pumpkin or psyllium husk, also exhibit improved functional properties and nutritional value, reinforcing the benefits of using alternative flours [32,33].

The oil absorption capacity (OAC) of the flours is influenced by their chemical composition [34]. The higher OAC of HMFP at 1.91 mL of oil/100 g compared to white wheat flour (1.46 mL) suggests that the fiber in HMFP enhances its ability to absorb oil. This phenomenon can be attributed to the structural properties of dietary fibers, which can create a network that retains oil more effectively through hydrophobic interactions and physical entrapment within the fiber matrix [35]. The incorporation of HMFP into white wheat flour resulted in a progressive increase in OAC from 1.46 mL to 1.57 mL per 100 g sample with varying HMFP levels (Table 1). This observed increase is consistent with previous studies reporting that fiber-rich ingredients tend to enhance oil absorption capacity through hydrophobic interactions and physical entrapment within the fiber matrix [32,35,36]. Studies on various flours, such as those blended with pumpkin or oat, show similar trends where higher fiber content correlates with increased OAC [32,34,36]. Specifically, Alija et al. [32] reported that the addition of pumpkin flour (5–15%) to wheat flour resulted in OAC increases from 1.48 to 1.63 mL oil/g sample, which is comparable to our observed increase from 1.46 to 1.57 mL oil/g sample with HMFP addition. In line with previous studies, composite flours with enhanced functional qualities have been reported to produce bread and chapattis with higher sensory evaluations [31,32,33,34,35,36,37]. The incorporation of HMFP resulted in a progressive increase in foaming capacity in composite flours, which is consistent with the presence of surface-active proteins and amphiphilic compounds that reduce surface tension at the air–water interface [29]. While foaming and emulsifying capacities are not directly utilized in Kleija production, these measurements provide insight into the protein functionality and surface-active properties of HMFP. The foaming capacity values ranged from 9.80% to 14.10%, with the highest foam capacity recorded for HMFP at 14.10%, while white wheat flour exhibited the lowest at 9.80%. The increase in foam capacity showed a dose-dependent pattern with HMFP concentration, with foam capacity increasing to 10.27% and 10.35% at 10% and 12% additions, respectively (Table 1). Emulsion activity (EA) of samples ranged from 10.50 to 40.10% (Table 1). The greatest EA was reported for HMFP, whereas the lowest was for white wheat flour. The emulsion activity of the composite flours improved as the HMFP concentration in the blends increased, likely due to the presence of soluble fiber and proteins that act as emulsifying agents by forming a stable interfacial film around oil droplets [35]. The EA increased from 10.50% in white wheat flour to 13.18 and 13.80% when 10 and 12% of HMFP were added, respectively (Table 1). These findings point to the unique chemical characteristics of HMFP, including its protein composition, fiber structure, and the presence of fermentation-derived surface-active metabolites, which have been previously reported for fermented sorghum products [16].

### 3.2. Chemical Composition Kleija Samples Incorporated with Different Quantities of Hulu-Mur Flakes Powder (HMFP)

The moisture content of Kleija products is crucial for determining their shelf life and quality. The observed moisture levels ranged from 14.20% to 14.70%, indicating a consistent moisture profile across samples, which is essential for maintaining product integrity (Table 2). These values are within the typical range reported for baked pastry products and are comparable to moisture contents of 14.2–15.1% reported in similar studies on traditional Saudi pastries [11,38]. Although the initial dough water content varied across formulations (from 5.0 L for the control to 6.14 L for 12% HMFP), the final baked products showed no significant differences in moisture content (14.20–14.70%; *p* > 0.05). This observation warrants careful consideration, as one might expect that the additional water added to HMFP-fortified doughs would result in higher final moisture content. Several factors may explain this apparent discrepancy. First, the higher water absorption capacity of HMFP (Table 1) indicates that the additional water was primarily bound within the fiber matrix rather than remaining as free water. During baking at 185 °C for 20 min, free water is readily evaporated, whereas bound water is retained within the fiber structure. The fact that the final moisture content remained constant despite higher initial water addition suggests that the additional water in HMFP-fortified samples was tightly bound to the fiber matrix and therefore not available for evaporation. This is consistent with the established mechanism of water binding by dietary fibers, where water molecules are immobilized through hydrogen bonding within the polysaccharide network [38]. Second, the baking process itself may have standardized moisture loss across formulations. The fixed baking time and temperature (185 °C for 20 min) ensured that all samples reached the same final internal temperature, which governs the extent of moisture evaporation. Once the internal temperature reached 100 °C, evaporation occurred until the vapor pressure equilibrated with the oven environment, resulting in similar final moisture contents regardless of initial water content. Third, the fiber matrix in HMFP may have formed a network that bound water more effectively, preventing excessive moisture loss during baking while also limiting the amount of free water available for retention. This dual effect would explain why the additional water added to HMFP-fortified doughs did not translate into higher final moisture content. It is also possible that the water adjustments made in this study (from 5.0 L to 6.14 L) were not sufficient to produce measurable differences in final moisture content, given the relatively small substitution levels (2–12% HMFP). The maximum difference in added water was 1.14 L (22.8% increase), which, after baking and evaporation, may have resulted in differences too small to detect statistically. The consistent final moisture content across all formulations is technologically advantageous, as it indicates that product quality and shelf-life potential remain uniform despite formulation differences [39,40]. However, further studies with higher substitution levels and more detailed analysis of water mobility (e.g., using nuclear magnetic resonance or differential scanning calorimetry) would be valuable to fully elucidate the water–fiber interactions in this system.

The incorporation of Hulu-mur flakes powder (HMFP) into Kleija samples resulted in a progressive increase in crude protein content, with values ranging from 14.70% to 15.60% (Table 2). As the levels of HMFP increase, the protein concentration in the Kleija samples also rises, showing a dose-dependent increase (*p* ≤ 0.05). Studies on Hulu-mur, a traditional Sudanese beverage made from fermented sorghum, indicate a protein content of around 14.0% in the flakes. Additionally, Hulu-mur is known to contain essential amino acids including valine, isoleucine, leucine, tyrosine, and phenylalanine [16,41]. The observed increase in protein content aligns with findings that high-protein ingredients can elevate the overall protein percentage in baked products [30,42,43,44]. Similar results have been reported by Prieto-Vazquez Del Mercado et al. [42], who found that protein-rich ingredients increased protein content in bread formulations by 1.5–2.0% at similar substitution levels. The fat content in developed Kleija samples, ranging from 19.84% to 20.42%, shows no significant differences (*p* > 0.05) when supplemented with varying levels of HMFP, which has a low fat content of 1.9% to 2.6% [41,45]. This indicates that the addition of HMFP does not substantially alter the overall fat percentage in Kleija, suggesting a stable fat profile regardless of supplementation (Table 2). These fat content values are comparable to those reported for traditional Kleija (19.5–20.5%) by Almuziere and Alhomaid [11]. The ash content of the Kleija samples ranged from 2.74% to 3.29%. Kleija samples with HMFP showed significantly higher ash content compared to the control samples, indicating that HMFP is a rich source of minerals. The highest recorded ash contents were 3.22% and 3.29% for samples with 10% and 12% HMFP, respectively, showing a progressive increase with HMFP concentration (Table 2). The increase in ash content of developed Kleija samples can be attributed to the high ash content of Hulu-mur flakes powder (HMFP), which enhances the mineral profile of the final product [41]. This observation is consistent with findings from previous studies [46,47], which reported that fortification of traditional snacks with ash-rich ingredients led to proportional increases in mineral content. Total dietary fiber in natural foodstuffs plays a crucial role in dietary benefits, including laxative effects and glycemic control [48]. The inclusion of Hulu-mur flakes powder (HMFP) in Kleija samples significantly enhanced their total dietary fiber content, with values rising from 6.12% in the control to 7.69% in samples containing 12% HMFP (Table 2). This progressive increase (a 1.26-fold improvement at the highest substitution level) is directly attributable to the high fiber content of HMFP, which is consistent with findings from other fiber-rich food additives [49,50]. These values are higher than those reported for conventional Kleija (4.5–5.2% dietary fiber) and are comparable to fiber-enriched bakery products containing 6.5–8.0% fiber [11,30]. Concomitantly, the total carbohydrate content (calculated by difference) decreased progressively with increasing HMFP substitution, ranging from 57.18% in the control to 54.18% in the 12% HMFP sample. This reduction reflects the displacement of wheat starch by the fiber-rich HMFP, which contains less available carbohydrates. This trend aligns with earlier reports that ingredient substitutions with high-fiber flours lower the carbohydrate fraction in baked goods [30,51]. The observed concurrent increases in protein, ash, and dietary fiber are consistent with similar studies on composite flour-based products. For example, Subramani et al. [31] reported proportional increases in protein (12–16%), ash (2.5–3.5%), and fiber (6–8%) when wheat flour was partially substituted with soybean and moringa flours. Similarly, Mamat et al. [29] observed comparable nutritional improvements in composite flour formulations containing up to 15% alternative protein and fiber sources, which are known to enhance overall nutritional value and physiological response [52,53,54,55,56,57,58,59,60]. The energy value of the Kleija samples showed a modest but significant decline, from 463.60 ± 5.30 kcal/100 g (control) to 459.14 ± 6.18 kcal/100 g at 12% HMFP (*p* ≤ 0.05). This decrease is a direct consequence of the lower available carbohydrate content and the replacement of starch (4 kcal/g) with dietary fiber (which contributes ~2 kcal/g), even though fat and protein levels remained largely stable. The energy reduction observed is consistent with studies on fiber-enriched baked products, where replacement of wheat flour with high-fiber ingredients typically results in 2–5% reductions in caloric density [30,51].

### 3.3. Mineral Content (mg/100 g) of Kleija Samples Incorporated with Different Quantities of Hulu-Mur Flakes Powder (HMFP)

Table 3 shows the mineral content of the control and HMFP-fortified Kleija samples.

The incorporation of HMFP resulted in progressive increases in all minerals studied with increasing substitution levels, showing a dose-dependent pattern (*p* ≤ 0.05) (Table 3). These essential dietary minerals are critical for supporting core physiological functions, metabolism, and human health [61,62,63,64,65,66,67,68,69,70,71,72,73,74,75,76,77,78,79,80,81]. Sodium (Na) content increased from 902.11 mg/100 g in the control sample to 1082.49 mg/100 g at 12% HMFP, representing a 1.20-fold increase. The significant differences (*p* ≤ 0.05) between substitution levels indicate a clear dose-dependent relationship. The sodium levels in the developed Kleija samples (902–1082 mg/100 g) are within the range reported for traditional baked pastries (850–1200 mg/100 g) [82]. Potassium (K) levels showed a progressive increase from 1144.78 mg/100 g (control) to 1384.81 mg/100 g (12% HMFP), representing a 1.21-fold increase. These values are comparable to those reported by Winiarska-Mieczan and Kwiecien [24] for fiber-enriched bakery products (1200–1400 mg/100 g). Calcium (Ca) concentrations increased significantly (*p* ≤ 0.05) from 150.02 mg/100 g (control) to 181.45 mg/100 g (12% HMFP). These values align with findings from Alija et al. [32], who recorded calcium levels of 140–190 mg/100 g in composite flour blends containing pumpkin flour. The 1.21-fold increase at 12% HMFP demonstrates the mineral enrichment potential of HMFP. Magnesium (Mg) showed a substantial increase from 400.33 mg/100 g (control) to 494.00 mg/100 g (12% HMFP), representing a 1.23-fold improvement. These values are consistent with studies on sorghum-based products, which typically contain 350–500 mg/100 g magnesium [41,45], and are higher than values reported for conventional wheat-based pastries (350–400 mg/100 g) [24]. Phosphorus (P) levels ranged from 28.91 mg/100 g (control) to 35.00 mg/100 g (12% HMFP), demonstrating a progressive increase. These values are consistent with the phosphorus content of fermented sorghum products (30–40 mg/100 g) reported by Mahgoub et al. [15]. Iron (Fe) content increased progressively from 9.73 mg/100 g (control) to 11.70 mg/100 g (12% HMFP), representing a 1.20-fold increase. This enrichment aligns with values reported for fortified cereal bars (10–12 mg/100 g) [83] and fermented cereal products (8–15 mg/100 g) [16,41]. Zinc (Zn) showed a progressive increase from 37.33 mg/100 g (control) to 44.43 mg/100 g (12% HMFP), consistent with zinc levels reported for fermented sorghum products (35–45 mg/100 g) [16,41]. Copper (Cu) content increased from 66.09 mg/100 g (control) to 78.88 mg/100 g (12% HMFP), within the range reported for sorghum-based products (60–80 mg/100 g) [41,45]. Manganese (Mn) increased progressively from 7.76 mg/100 g (control) to 9.26 mg/100 g (12% HMFP), consistent with manganese content reported for fermented cereal products (7–10 mg/100 g) [24,41]. The observed increases in mineral content are attributed to the mineral richness of HMFP, which enhanced the overall mineral profile of the fortified Kleija compared to control samples. These results are consistent with previous studies showing that incorporating fiber- and mineral-rich ingredients into bakery products improves the final product’s mineral content [30,32,47]. The significant dose-dependent increases (*p* ≤ 0.05) for all minerals confirm that HMFP is an effective source of essential minerals, particularly micro-minerals that contribute significantly to cellular metabolism, oxygen transport, and physiological regulation [84,85,86,87].

### 3.4. Bioactive Compounds and Antioxidant Activity of Kleija Samples Fortified with Varying Levels of HMFP

Table 4 shows the total phenolics, total flavonoids, total carotenoids and DPPH radical scavenging activity % of Kleija samples incorporated with different quantities of HMFP.

The incorporation of HMFP into the Kleija samples resulted in significant (*p* ≤ 0.05) dose-dependent increases in total phenolic, flavonoid, and carotenoid contents. The observed increases in bioactive compounds and antioxidant activity are directly attributable to the addition of HMFP, which is rich in these compounds [14,16], rather than to synergistic interactions. The total phenolic content increased from 700.06 mg GAE/100 g DW in the control sample to 1698.44 mg GAE/100 g DW at 12% HMFP, representing a 2.42-fold increase. Total flavonoids increased from 228.90 to 534.81 mg CE/100 g DW (2.33-fold increase), while total carotenoids increased from 5.48 to 8.22 mg/g (1.50-fold increase). Correspondingly, DPPH radical scavenging activity increased from 44.86% to 69.13% at 12% HMFP, representing a 1.54-fold improvement. This enhancement is reflected in the substantial increase in DPPH radical scavenging activity, indicating the effectiveness of HMFP in improving the antioxidant properties of Kleija. This behavior is attributed to the key role of phenolic compounds in enhancing free radical scavenging capacity [88,89]. Studies indicate that phenolic compounds are key contributors to antioxidant activity, with a strong relationship between their concentration and the ability to scavenge free radicals [90,91,92,93]. These values are consistent with previously reported ranges for fermented sorghum products. Salih et al. [14] reported total phenolic content ranging from 305.17 to 1600 mg GAE/100 g in fermented sorghum products, with fermentation significantly increasing phenolic content compared to raw flour. Abdelhalim et al. [16] reported flavonoid contents of 400–550 mg CE/100 g and DPPH scavenging activity of 60–75% in Hulu-mur beverages prepared from different sorghum landraces. The total carotenoid values recorded (5.48–8.22 mg/g) are within the range reported for sorghum-based products (5–10 mg/g) [94,95,96]. The progressive increase in bioactive compounds and antioxidant activity with higher HMFP levels is consistent with the known composition of fermented sorghum products. The progressive increase in bioactive compounds and antioxidant activity with higher HMFP levels is consistent with the known composition of fermented sorghum products [14,16]. The increased bioactive compound content, particularly phenolic compounds, flavonoids, and carotenoids, contributes to the enhanced DPPH radical scavenging activity through their ability to donate hydrogen atoms or electrons to neutralize free radicals, thereby protecting against cell damage and reactive oxygen species [97,98,99,100,101]. These results align with findings from other studies on fortified bakery products. Guiné and Florença [9] reported that incorporation of bioactive-rich ingredients into bakery products resulted in 1.5–2.5-fold increases in total phenolic content and corresponding improvements in antioxidant activity. Similarly, Mohammed et al. [28] observed dose-dependent increases in phenolic content and DPPH scavenging activity when bread was fortified with cheeseweed mallow leaves powder.

### 3.5. Sensory Evaluation of Kleija Samples Incorporated with Different Quantities of Hulu-Mur Flakes Powder (HMFP)

**Figure 3:**
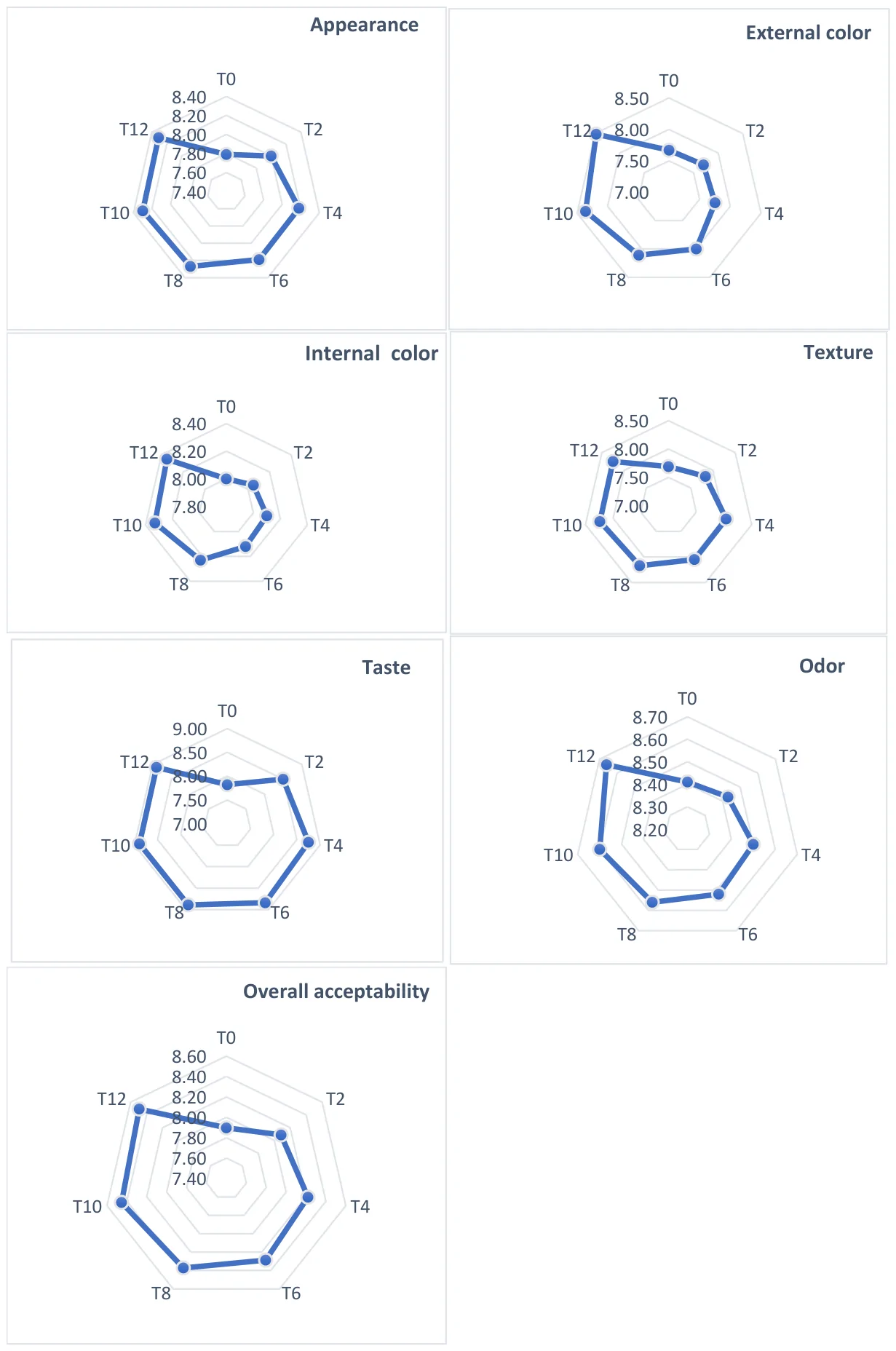
Sensory evaluation of Kleija samples incorporated with different quantities of Hulu-mur flakes powder (HMFP).

The sensory properties of food, including taste, texture, odor, and appearance, significantly influence consumer acceptability and eating behaviors [89]. The sensory properties of Kleija samples incorporated with different quantities of HMFP are presented in Figure 3.

The incorporation of HMFP resulted in progressive increases in all sensory attribute scores with increasing substitution levels up to 12%. The overall acceptability scores ranged from 7.48 (control) to 8.49 (12% HMFP), with samples containing 8%, 10%, and 12% HMFP achieving scores of 8.37, 8.45, and 8.49, respectively. This dose-dependent improvement in acceptability suggests that HMFP positively influenced consumer perception across multiple sensory dimensions. Appearance and color attributes showed the most consistent improvements with HMFP incorporation.

Appearance and color attributes showed the most consistent improvements with HMFP incorporation. Appearance scores increased from 7.79 (control) to 8.31 (12% HMFP), underscoring the role of visual attributes as a primary interaction point in driving consumer preference [102], while exterior color increased from 7.62 to 8.48 and interior color from 7.55 to 8.35. The highest scores for these attributes were consistently observed at 8–12% HMFP substitution levels. These improvements can be attributed to the characteristic golden-brown color imparted by HMFP, which consumers found more appealing than the lighter color of the control sample. A positive appearance can enhance perceptions of other sensory attributes, such as flavor and texture [103]. This finding is consistent with previous reports that pigmented flours enhance visual appeal and consumer preference in baked products [104]. Texture and taste attributes also showed progressive improvements with increasing HMFP levels. Texture scores increased from 7.69 (control) to 8.25 (12% HMFP), with samples containing 8%, 10%, and 12% HMFP achieving scores of 8.17, 8.24, and 8.25, respectively. Taste scores showed the most pronounced enhancement, increasing from 7.82 (control) to 8.90 (12% HMFP), with the highest scores consistently observed at 8–12% HMFP. The high taste scores indicate that HMFP supplementation positively influenced the flavor profile without introducing off-flavors, a finding consistent with previous reports on fermented sorghum products [16]. These results are also consistent with studies on muffins and other baked products where fiber-rich ingredients enhanced texture and sensory attributes at optimal substitution levels [105]. Odor scores followed a similar pattern, increasing progressively from 7.48 (control) to 8.66 (12% HMFP), with the highest score recorded at 12% HMFP. The variation in odor scores highlights the significant impact of ingredient chemical composition on sensory properties [106]. This suggests that HMFP contributed desirable aromatic compounds to the Kleija, likely due to the unique fermentation-derived volatile compounds present in the sorghum-based ingredient [14,16]. The overall acceptability scores for all HMFP-fortified samples exceeded 7.5 (on a 9-point scale), confirming that consumers accepted the modified products and generally preferred them to the control. These results are consistent with previous studies on sensory evaluation of fortified bakery products. Almuziere and Alhomaid [11] reported acceptability scores of 7.2–8.5 for Kleija formulated with date-based sweeteners, while Barakat et al. [12] reported similar scores for Kleija-like biscuits formulated with sprouted mung beans. The acceptability scores observed in this study (7.48–8.49) are comparable to these reported ranges. In summary, the dose-dependent improvement in sensory scores up to 12% HMFP demonstrates that HMFP incorporation does not negatively affect sensory quality and may actually enhance consumer preference. However, further studies with substitution levels exceeding 12% are warranted to determine whether product quality continues to improve or whether an optimal threshold exists beyond which sensory acceptability may decline.

### 3.6. Heat Map of a Correlation Matrix

**Figure 4:**
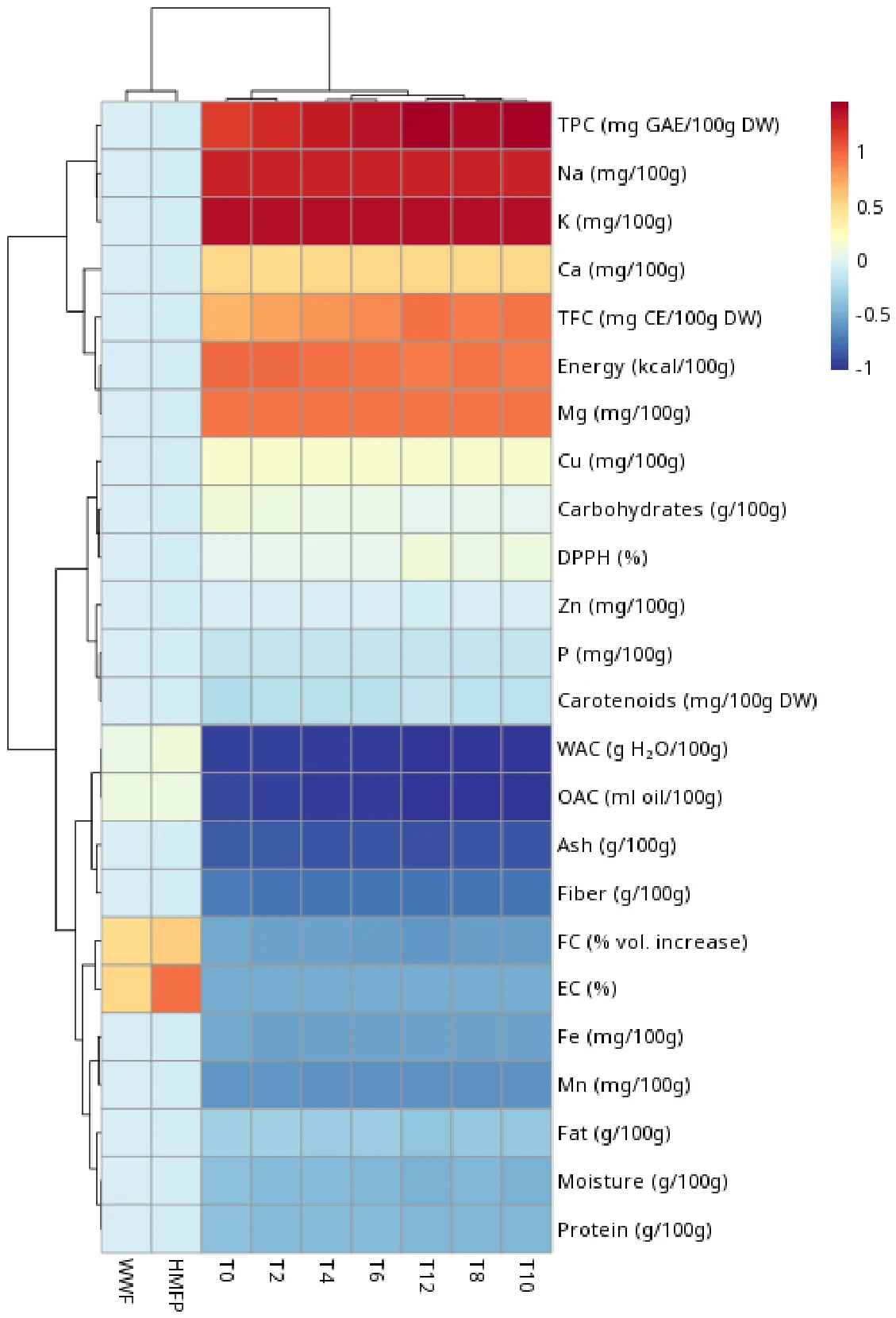
Correlation heat map of Hulu-mur flakes powder (HMFP) incorporation levels with the nutritional composition, mineral content, bioactive compounds, and antioxidant activity of fortified Kleija samples.

The heat map of a correlation matrix (Figure 4) summarizes the results of the current investigation. This heat map concluded that the incorporation of HMFP into wheat flour during Kleija preparation significantly enhances its nutritional profile, particularly in protein, fat, ash, and fiber content. However, this substitution leads to a notable decrease in total carbohydrates and energy values. Additionally, the mineral content of the resulting Kleija products improves, alongside increases in total phenolics, flavonoids, carotenoids, and antioxidant activity, as measured by DPPH radical scavenging percentage.

## 4. Conclusions

This study successfully developed a novel, nutritionally enhanced version of the traditional Saudi pastry, Kleija, by incorporating Sudanese Hulu-mur flakes powder (HMFP) at substitution levels of up to 12% (*w*/*w*). The findings demonstrate that this approach significantly improves the nutritional and functional properties of the product without compromising its sensory acceptability.

The incorporation of HMFP led to substantial enhancements in the bioactive profile of Kleija, with 2.42-fold and 2.33-fold increases in total phenolics and flavonoids, respectively, and a 1.54-fold boost in antioxidant activity at the 12% substitution level. These results confirm that HMFP is an effective functional ingredient that can significantly alter the product’s biochemical composition. Additionally, the research elucidated the techno-functional rationale behind these improvements, linking the high-fiber and unique chemical composition of HMFP to its superior water/oil absorption, emulsifying, and foaming capacities, which in turn influenced the dough properties and final product quality.

From a practical perspective, this research provides a clear formulation for creating a Kleija variant that is richer in dietary fiber, plant-based protein, and essential minerals (such as iron, zinc, and magnesium), while simultaneously reducing its carbohydrate content and energy value. The high consumer acceptability scores, particularly for samples with 8–12% HMFP, indicate strong market potential and demonstrate the viability of this approach for commercialization. This work successfully demonstrates a model for dietary supplementation, offering a way to bridge nutritional gaps by innovating within the framework of culturally significant and widely accepted ethnic foods. It positions the developed HMFP–Kleija not just as a traditional snack, but as a nutritionally enhanced product capable of contributing to a health-conscious diet while preserving cultural food identity.

Future research should focus on several critical areas to advance this work. First, in vivo bioavailability studies are needed to confirm that the enhanced nutrient and antioxidant levels translate to improved biological markers in human subjects. Second, shelf-life stability investigations should be conducted to determine the oxidative stability of the fortified product over time. Third, optimization of HMFP processing conditions could maximize bioactive compound retention while maintaining techno-functional properties. Fourth, further studies with substitution levels exceeding 12% are warranted to determine the maximum feasible HMFP incorporation level before sensory quality deterioration occurs. Finally, exploration of other traditional grain products as potential functional ingredients in various food matrices would help establish general principles for this approach.

## Figures and Tables

**Table 1:** Techno-functional properties of white wheat flour, Hulu-mur flakes powder (HMFP) and composite flours.

Functional Property	WWF	HMFP	HMFP Addition Ratio (Substitution %)					
			2%	4%	6%	8%	10%	12%
WAC (g water/g sample)	1.40 ^d^ ± 0.11	2.33 ^a^ ± 0.09	1.42 ^d^ ± 0.10	1.44 ^c^ ± 0.09	1.46 ^c^ ± 0.12	1.49 ^b^ ± 0.11	1.51 ^b^ ± 0.07	1.53 ^b^ ± 0.07
OAC (mL oil/g sample)	1.46 ^d^ ± 0.10	1.91 ^a^ ± 0.13	1.47 ^d^ ± 0.09	1.48 ^d^ ± 0.10	1.50 ^c^ ± 0.10	1.51 ^c^ ± 0.07	1.54 ^bc^ ± 0.06	1.57 ^b^ ± 0.07
FC (% vol. increase)	9.80 ^e^ ± 0.43	14.10 ^a^ ± 0.77	9.92 ^d^ ± 0.53	9.99 ^d^ ± 0.61	10.10 ^cd^ ± 0.32	10.18 ^c^ ± 0.40	10.27 ^bc^ ± 0.22	10.35 ^b^ ± 0.16
EA (%)	10.50 ^e^ ± 0.36	40.10 ^a^ ± 0.97	10.79 ^e^ ± 0.26	11.38 ^d^ ± 0.71	12.00 ^cd^ ± 0.50	12.60 ^c^ ± 0.66	13.18 ^b^ ± 0.41	13.80 ^b^ ± 0.33

Values are means ± SD of five determinations. Means in the same row with different letters are significantly different (*p* ≤ 0.05). WWF means white wheat flour; other terms are water absorption capacity (WAC), oil absorption capacity (OAC), emulsion activity (EA), and foam capacity (FC).

**Table 2:** Chemical composition Kleija samples incorporated with different quantities of Hulu-mur flakes powder (HMFP).

Components (g/100 g Dry Weight Basis)	0% HMFP	2% HMFP	4% HMFP	6% HMFP	8% HMFP	10% HMFP	12% HMFP
Moisture	14.20 ^a^ ± 0.56	14.34 ^a^ ± 0.67	14.48 ^a^ ± 0.78	14.61 ^a^ ± 0.84	14.65 ^a^ ± 0.58	14.68 ^a^ ± 0.77	14.70 ^a^ ± 0.58
Crude protein	14.70 ^a^ ± 0.89	14.86 ^a^ ± 0.98	14.97 ^ab^ ± 0.89	15.17 ^b^ ± 0.31	15.29 ^b^ ± 0.67	15.44 ^b^ ± 0.87	15.60 ^c^ ± 0.59
Fat content	19.84 ^a^ ± 0.56	19.90 ^a^ ± 0.87	20.04 ^a^ ± 0.83	20.10 ^a^ ± 0.87	20.18 ^a^ ± 0.94	20.25 ^a^ ± 0.76	20.42 ^a^ ± 0.56
Ash	2.74 ^a^ ± 0.084	2.85 ^ab^ ± 0.09	2.92 ^b^ ± 0.07	3.05 ^c^ ± 0.04	3.13 ^d^ ± 0.05	3.22 ^de^ ± 0.03	3.29 ^e^ ± 0.02
Total dietary fibers	6.12 ^a^ ± 0.18	6.48 ^b^ ± 0.21	6.74 ^c^ ± 0.16	6.93 ^d^ ± 0.19	7.18 ^e^ ± 0.14	7.45 ^f^ ± 0.17	7.69 ^g^ ± 0.15
Total carbohydrates	57.18 ^d^ ± 2.31	56.58 ^c^ ± 2.87	56.07 ^bc^ ± 3.45	55.54 ^b^ ± 2.87	55.12 ^ab^ ± 2.76	54.63 ^a^ ± 3.31	54.18 ^a^ ± 3.23
Available carbohydrates (total CHO-TDF) (%)	51.06 ^d^ ± 2.32	50.10 ^cd^ ± 2.88	49.33 ^cb^ ± 3.45	48.61 ^b^ ± 2.88	47.94 ^ab^ ± 2.76	47.18 ^a^ ± 3.31	46.49 ^a^ ± 3.24
Energy value (kCal/100 g)	463.60 ^d^ ± 5.30	462.14 ^cd^ ± 5.98	461.56 ^cb^ ± 4.76	460.82 ^b^ ± 6.58	460.34 ^ab^ ± 5.15	459.53 ^a^ ± 4.32	459.14 ^a^ ± 6.18

Values are means ± SD of five determinations. Means in the same row with different letters are significantly different (*p* ≤ 0.05). Available Carbohydrate = Total Carbohydrate − Total Dietary Fiber. Energy Value = (Protein × 4) + (Available Carbohydrate × 4) + (Fat × 9). HMFP0 represents control samples; HMFP 2, HMFP 4, HMFP 6, HMFP 8, HMFP 10 and HMFP 12 refer to developed Kleija samples where wheat flour was partially substituted with Hulu-mur flakes powder at 2, 4, 6, 8, 10 and 12% (*w*/*w*), respectively.

**Table 3:** Mineral content (mg/100 g) of Kleija samples incorporated with different quantities of Hulu-mur flakes powder (HMFP).

Mineral Analysis (mg/100 g)	0% HMFP	2% HMFP	4% HMFP	6% HMFP	8% HMFP	10% HMFP	12% HMFP
Na	902.11 ^a^ ± 5.98	937.88 ^ab^ ± 6.72	956.18 ^b^ ± 6.87	1001.26 ^c^ ±4.98	1028.38 ^d^ ± 7.99	1064.68 ^de^ ± 8.95	1082.49 ^e^ ± 9.62
K	1144.78 ^a^ ± 6.96	1173.42 ^b^ ± 8.90	1219.19 ^c^ ± 6.89	1282.15 ^d^ ± 7.87	1293.71 ^e^ ± 8.65	1352.01 ^f^ ± 8.63	1384.81 ^g^ ± 7.56
Ca	150.02 ^a^ ± 2.23	153.02 ^ab^ ± 2.54	159.92 ^b^ ± 3.12	166.82 ^c^ ± 3.65	172.21 ^d^ ± 2.69	175.67 ^de^ ± 3.67	181.45 ^e^ ± 3.89
Mg	400.33 ^a^ ±4.67	416.74 ^b^ ± 5.76	426.37 ^bc^ ± 4.87	446.00 ^c^ ± 6.41	456.23 ^cd^ ± 4.65	468.88 ^d^ ± 5.53	494.00 ^e^ ± 4.52
*p*	28.91 ^a^ ± 1.21	29.94 ^ab^ ± 2.31	30.62 ^ab^ ± 1.76	32.31 ^b^ ± 1.46	32.98 ^b^ ± 2.34	33.87 ^bc^ ± 1.43	35.00 ^c^ ± 2.45
Fe	9.73 ^a^ ± 0.87	10.06 ^a^ ± 0.56	10.33 ^ab^ ± 0.87	10.80 ^b^ ± 0.98	11.12 ^bc^ ± 0.49	11.46 ^c^ ± 0.89	11.70 ^c^ ± 0.99
Zn	37.33 ^a^ ± 2.22	39.00 ^b^ ± 2.31	39.48 ^b^ ± 3.54	41.43 ^c^ ± 3.31	42.50 ^cd^ ± 2.87	43.70 ^d^ ± 2.47	44.43 ^e^ ± 3.52
Cu	66.09 ^a^ ± 3.76	68.70 ^b^ ± 4.51	70.00 ^c^ ± 3.88	73.35 ^d^ ± 4.69	75.37 ^e^ ± 4.37	77.30 ^f^ ± 5.61	78.88 ^g^ ± 4.98
Mn	7.76 ^a^ ± 0.38	8.05 ^b^ ± 0.37	8.20 ^b^ ± 0.87	8.57 ^bc^ ± 0.54	8.87 ^c^ ± 0.87	9.09 ^d^ ± 0.76	9.26 ^de^ ± 0.84

Values are means ± SD of five determinations. Means in the same row with different letters are significantly different (*p* ≤ 0.05). HMFP0 represents control samples; HMFP 2, HMFP 4, HMFP 6, HMFP 8, HMFP 10 and HMFP 12 refer to developed Kleija samples where wheat flour was partially substituted with Hulu-mur flakes powder at 2, 4, 6, 8, 10 and 12% (*w*/*w*), respectively.

**Table 4:** Total phenolics, total flavonoids, total carotenoids and DPPH radical scavenging activity % of Kleija samples incorporated with different quantities of Hulu-mur flakes powder (HMFP).

Parameter	0% HMFP	2% HMFP	4% HMFP	6% HMFP	8% HMFP	10% HMFP	12% HMFP
Total phenolics (mg GAE/100 g DW)	700.06 ^a^ ± 7.78	867.41 ^b^ ± 8.86	1043.91 ^c^ ± 8.74	1225.80 ^d^ ± 9.89	1402.66 ^e^ ± 7.88	1580.12 ^f^ ± 7.98	1698.44 ^g^ ± 8.94
Total flavonoid mg catechin equivalents (CE)/100 g DW	228.90 ^a^ ± 3.41	278.94 ^b^ ± 4.76	327.88 ^c^ ± 5.69	377.85 ^d^ ± 6.31	431.73 ^e^ ± 5.87	473.07 ^f^ ± 5.33	534.81 ^g^ ± 5.49
Total carotenoids (mg/ 100 g DW)	24.66 ^a^ ± 0.67	26.71 ^ab^ ± 0.89	27.08 ^b^ ± 0.74	28.92 ^bc^ ± 0.84	31.52 ^c^ ± 0.77	33.74 ^d^ ± 0.49	36.90 ^e^ ± 0.78
DPPH radical scavenging activity %	44.81 ^a^ ± 0.79	48.91 ^b^ ± 0.85	51.44 ^c^ ± 0.61	55.11 ^d^ ± 0.87	58.15 ^e^ ± 0.76	63.18 ^f^ ± 0.87	69.13 ^g^ ± 0.59

Values are means ± SD of five determinations. Means in the same row with different letters are significantly different (*p* ≤ 0.05). HMFP0 represents control samples; HMFP 2, HMFP 4, HMFP 6, HMFP 8, HMFP 10 and HMFP 12 refer to developed Kleija samples where wheat flour was partially substituted with Hulu-mur flakes powder at 2, 4, 6, 8, 10 and 12% (*w*/*w*), respectively.

## Data Availability

The original contributions presented in this study are included in the article. Further inquiries can be directed to the corresponding author.
